# Distribution Characteristics and Influencing Factors of Central Apnea in Chinese Pediatric Patients With Obstructive Sleep Apnea: A Single-Center Study

**DOI:** 10.3389/fped.2022.882352

**Published:** 2022-07-12

**Authors:** Jing Liu, Li Chang, Ling Cao, Guimin Huang

**Affiliations:** ^1^Department of Respiratory Medicine, Children's Hospital Affiliated to Capital Institute of Pediatrics, Beijing, China; ^2^Graduate School of Peking Union Medical College, Chinese Academy of Medical Sciences, Beijing, China; ^3^Child Health Big Data Research Center, Capital Institute of Pediatrics, Beijing, China

**Keywords:** pediatric, obstructive sleep apnea, central apnea, polysomnography, OSA

## Abstract

**Background:**

Central apnea (CA) events always can be seen in the polysomnographic (PSG) reports of children with obstructive sleep apnea (OSA), and sometimes the central apnea index (CAI) is higher than the obstructive apnea and hypopnea index (OAHI). Commonly, the clinicians only attribute it to the age. This study aims to elucidate the distribution characteristics and major factors associated with CA in pediatric OSA.

**Methods:**

A retrospective chart review of PSG data of children with OSA from January 2017 to March 2018 was performed.

**Results:**

856 children (317 girls and 539 boys, 4.9 ± 2.4 years) were involved. 50.1% (429/856) had a CAI > 1, and 2.9% (25/856) had a CAI >5. Children with a CAI >1 had a higher OAHI, arousal index (AI), oxygen desaturation index (ODI), and a longer REM period, but a younger age and a shorter slow-wave sleep (SWS) phase. Multivariate binary logistic regression showed that with a 1% increased REM period, the risk of the CAI being >1 increased by 5.3% (*p* < 0.001). The CAI increased with an increasing OAHI (*p* = 0.003). The possibility of a CAI ≤ 1 increased with age (*p* < 0.001), and boys were more likely to have a CAI ≤ 1 (*p* = 0.001).

**Conclusions:**

In addition to obstructive apnea (OA), almost all children with OSA also had CA, and a CAI > 1 was most likely to occur. The OAHI and REM period were risk factors for an increased CAI, and age and male sex were protective factors.

## Introduction

The sleep related breathing disorders are characterized by respiratory disorders during sleep and some daytime symptoms, such as snoring, mouth breathing, behavioral and learning issues, which can lead to serious consequences if unidentified and untreated (1, 2). The 3rd edition of the International Classification of Sleep Disorders (ICSD-3) grouped these disorders into obstructive sleep apnea (OSA), central sleep apnea (CSA) syndromes, sleep related hypoventilation and sleep related hypoxemia (3). In the pediatric population, OSA is the disease with the highest incidence, the most complications, and the most harmful among sleep related breathing disorders, which has received extensive attention (4–6). The prevalence of CSA is much lower than that of OSA. CSA is usually related to underlying disease states, such as congenital craniofacial malformations, brainstem, and spinal cord diseases, genetic conditions (7–11).

The diagnosis is usually based on the dominant respiratory disorders of polysomnography (PSG) (12). The main respiratory events in children with OSA are obstructive, in which the obstructive apnea hypopnea index (OAHI) is an index to reflect the severity. Central apnea index (CAI) ≥ 5 is considered to be the diagnostic standard of PSG for CSA (3). Central apnea (CA) events occur occasionally in children during sleep, including healthy children and children with OSA or other diseases (13–15). However, the significance of CA events is unclear. It has been reported that CA events occur in healthy children at the beginning of sleep, after awakening, or after sighing, but they are generally considered physiological, the frequency is usually very low, and are not scored by PSG (16). In our clinic, we found that CA events always can be seen in the PSG reports of children with OSA, and sometimes it is difficult to diagnose when central apnea index (CAI) is higher than OAHI. There are few studies on the occurrence of CA events in children with OSA. At present, the characteristics of CA, the threshold of CAI, and the prevalence of CSA in children with OSA are not clear.

The pathogenesis and clinical manifestations of central apnea and obstructive apnea are different. The current research evidences have not clarified whether the pathophysiological characteristics of children with OSA affect the stability of respiratory central ventilation, which leads to the occurrence of central events. We hypothesized that there may be a certain correlation between central apnea and obstructive apnea, and some factors may drive the occurrence of central apnea in pediatric OSA. The purpose of this study is to summarize the distribution characteristics and influencing factors of CA events in children with OSA to guide clinical practice.

## Methods

### Patients

A retrospective chart review of polysomnographic data of children diagnosed with OSA from January 2017 to March 2018 was performed. All patients had at least one sleep complaint, such as snoring, apnea, open-mouth breathing, or daytime sleepiness, and underwent PSG in the Sleep Respiration Monitoring Center in our hospital. OSA was diagnosed based on the criteria of the ICSD-3. Patients were classified into mild (1 ≤ OAHI < 5), moderate (5 ≤ OAHI < 10) and severe groups (OAHI ≥ 10) (17). To study and identify the influencing factors of central apnea events in children with OSA, we divided them into two groups (CAI ≤ 1 and CAI > 1). Children with one or more of the following conditions were excluded: central sleep apnea syndrome (CSAS) (caused by medical or neurological disorders, medication use, substance use, and other factors), neuropsychiatric or neuromuscular disorders, craniofacial dysplasia, a clear history of gastroesophageal reflux, heart failure, hypertension, hypothyroidism, long-term use of sleep aids or other psychotropic drugs, a history of prematurity, systemic therapy (CPAP treatment for more than 1 month), anti-inflammatory drug treatment, adenoidectomy or tonsillectomy.

### Overnight Sleep Studies

Children enrolled in this study were subjected to overnight PSG (Philips Alice 6, LDXS, USA), including electroencephalography (EEG), left and right electrooculography (EOG), submental electromyography (EMG), snoring recording, body position recording, finger pulse oximetry (SpO2), electrocardiography (ECG), heart rate recording, and thoracoabdominal movement measurement by thoracic and abdominal effort belts. Nasal air flow was recorded utilizing a thermistor and a pressure nasal cannula. Respiratory effort was measured by respiratory inductance plethysmography. According to the guidelines of the AASM (18), apnea was defined as a drop in the peak airflow by at least 90% of baseline lasting at least two respiratory cycles, (1) if the respiratory effort is sustained, apnea was considered as obstructive; (2) If there is no relevant respiratory effort during the whole event, lasting for at least 20 s or two respiratory cycles that is associated with either an SaO2 drop of ≥3% or an arousal, it is judged as central apnea; (3) If there was no respiratory effort during one part of the event but there was respiratory effort during another part, regardless of which occurred first, apnea was considered mixed. Hypopnea was defined as a reduction in the nasal cannula airflow >30% that lasted ≥90% of the duration of at least two normal breaths, accompanied by ≥3% oxyhemoglobin desaturation or arousal. Hypopneas can be classified as obstructive if any of the following criteria are met: snoring, flattening of nasal pressure signal, or paradoxical thoracoabdominal breathing. All PSG findings were scored by two experienced pediatric sleep specialists.

The main indicators and definitions are as follows. The obstructive apnea index (OAI) and central apnea index (CAI) were computed as the number of obstructive apnea events and the number of central apnea events per hour of sleep, respectively. The OAHI was defined as the sum of obstructive apnea events, mixed apnea events and obstructive hypopnea events per hour of sleep. The oxygen desaturation index (ODI) was defined as the number of hourly decreases in oxygen saturation exceeding 3%. Min Sat/Mean Sat was defined as the minimum/average percutaneous oxygen saturation (SpO2) during night sleep.

### Data Analysis

Data of continuous quantitative variables are represented as the means ± standard deviations. The Kolmogorov-Smirnov test was used to determine whether the distribution of all variables was normal. All the baseline characteristics of the subject population were not normally distributed; therefore, non-parametric statistical analysis was used. The chi-square test and Kruskal-Wallis test were used to analyze the differences in variables among the mild, moderate and severe groups, and the Mann-Whitney test was used to analyze the differences in continuous variables between the CAI ≤ 1 group and the CAI >1 group. The population distribution among the different degrees of CAI is displayed graphically. Associations between the CAI and potential possible influencing factors were evaluated by Spearman rank-order correlation analysis, and a significant correlation was considered at 0.40 or higher. The independent variables with *p* < 0.05 were screened by univariate binary logistic regression analysis. We eliminated the variables with collinearity problems and ultimately included age, sex (reference = girl), rapid eye movement (REM) period, slow-wave sleep (SWS) period, and OAHI in the multivariate logistic regression analysis. Considering CAI as the dependent variable, age, sex (1 = boy), REM period, SWS period, and the OAHI were considered covariables. Using the forward-stepwise method, in which the probability for conditional entry of predictors was 0.05 and that for the removal of predictors was 0.10, The final model contains all the factors significantly related to the results. All tests were two-tailed, and differences were considered statistically significant at *p* < 0.05. IBM SPSS Statistics 24.0 was used for the statistical analyses.

## Results

### Patients

In total, 856 children were eligible for inclusion; there were 317 girls and 539 boys, with a mean age of 4.9 ± 2.4 years. There were 374 mild cases, 261 moderate cases, and 221 severe cases. The baseline characteristics are shown in Table 1. The moderate and severe groups had higher CAIs, higher AIs, lower Min Sats and higher ODIs than the mild group. Although the average ages of the moderate and severe groups were not different, the severe group had a shorter REM period and a very significantly higher ODI than the other groups. There were no differences in the sex ratio or SWS period among groups.

**Table 1:** Demographic and sleep parameter differences between the mild, moderate and severe pediatric OSA groups.

**Variable**	**Mild (1 ≤OAHI <5)**	**Moderate (5 ≤OAHI <10)**	**Severe (OAHI ≥ 10)**	**Total patients**	**χ2/H**	***p*-value**
Age (years)	5.3, 2.6	4.7, 2.3	4.7, 2.4	4.9, 2.4	11.450	0.003
Boy/Girl	226/148	163/98	150/71	539/317	3.345	0.188
CAI	1.1, 1.0	1.7, 1.5	1.9, 2.0	1.5, 1.5	36.623	<0.001
Min Sat (%)	90.5, 4.3	86.4, 8.9	82.9, 10.8	87.3, 8.5	178.378	<0.001
Mean Sat (%)	97.9, 1.2	97.7, 1.0	97.1, 1.5	97.6, 1.3	69.373	<0.001
ODI	2.2, 1.2	5.1, 2.6	17.3, 16.5	7.0, 10.6	507.178	<0.001
TST (minutes)	405.2, 54.2	416.4, 53.1	401.7, 57.8	407.8, 55.1	6.829	0.063
LS (%)	27.7, 19.8	31.2, 16.1	32.6, 16.1	30.1, 17.9	7.073	0.029
SWS (%)	56.8, 21.0	54.5, 18.3	54.6, 18.7	55.6, 19.6	1.206	0.547
REM (%)	15.3, 6.4	14.3, 6.8	12.6, 7.8	14.4, 7.0	21.526	<0.001
AI	0.5, 0.4	1.4, 1.2	3.3, 2.6	1.5, 1.8	491.147	<0.001

*CAI, central apnea index; Min Sat, minimum oxygen saturation; Mean Sat, mean oxygen saturation; ODI, oxygen desaturation index; LS, light seep; SWS, slow-wave sleep; REM, rapid eye movement; AI, arousal index; TST, total sleep time*.

### Distribution of CAIs in Children With OSA

**Figure 1:**
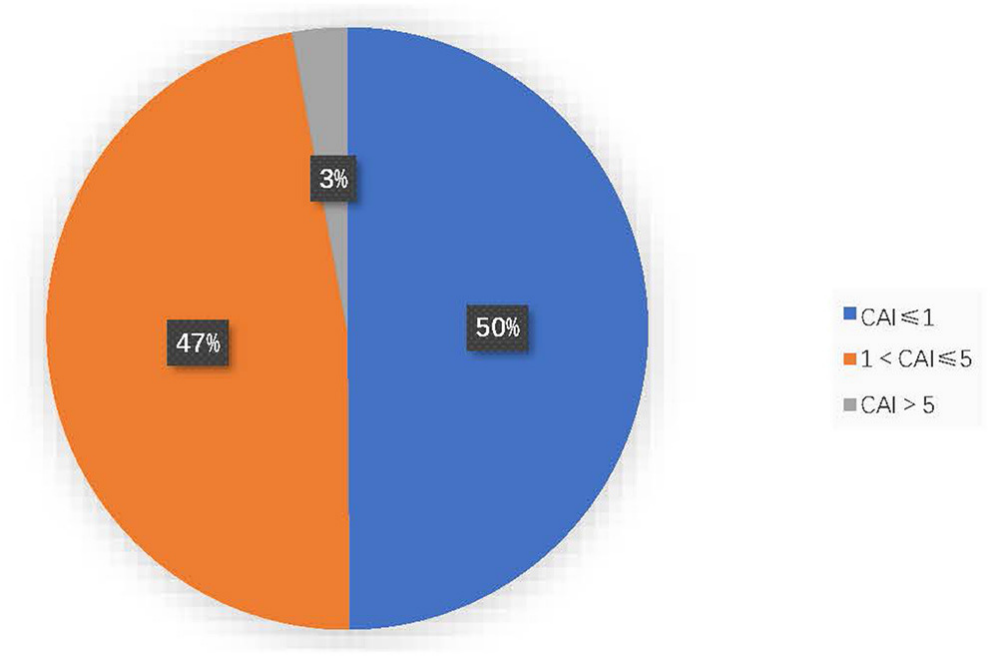
Distribution of different degrees of CAI in children with OSA.

**Figure 2:**
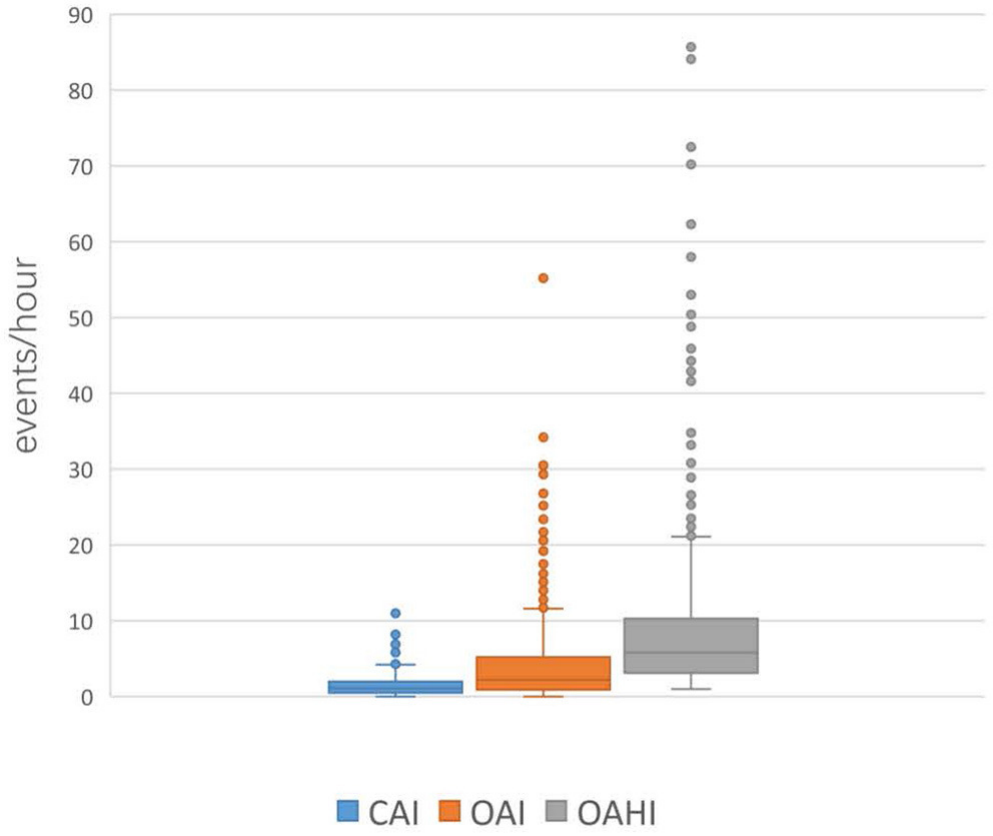
Distribution of the CAI, OAI, and OAHI in all children with OSA.

Among all the included OSA patients, 50.1% (429/856) had a CAI > 1, and 2.9% (25/856) had a CAI >5. CAI distribution proportions are presented in Figure 1. In the 856 subjects, the CAI ranged from 0 to 11.1, the OAHI ranged from 1 to 85.7, and the OAI ranged from 0 to 55.2. Accordingly, the median OAHI was 5.8 compared with the median CAI, at 1.1 (Figure 2). In contrast with the Mean Sat and light sleep (LS) period, there were significant differences in age, sex, OAHI, AI, OAI, Min Sat, ODI, SWS period and REM period between the CAI ≤ 1 group and the CAI > 1 group (Table 2). The CAI > 1 group had a higher OAHI (*p* < 0.001), AI (*p* < 0.001), OAI (*p* < 0.001), and ODI (*p* < 0.001); and a longer REM period (*p* < 0.001) but a younger age (*p* < 0.001); lower Min Sat (*p* = 0.019); and shorter SWS period (*p* = 0.016) than the CAI ≤ 1 group.

**Table 2:** Demographic and PSG characteristic differences between the CAI ≤ 1 and CAI >1 pediatric OSA groups.

**Variable**	**CAI ≤1 group**	**CAI > 1 group**	***p*-value**
Age (years)	5.4, 2.7	4.5, 2.1	<0.001
Boy/Girl	286/141	253/176	0.015
OAHI	7.9, 9.7	9.6, 10.1	<0.001
OAI	3.2, 4.7	4.6, 5.1	<0.001
Min Sat (%)	87.5, 8.9	87.1, 8.1	0.019
Mean Sat (%)	97.7, 1.2	97.6, 1.5	0.866
ODI	6.4, 9.4	7.7, 11.5	<0.001
LS (%)	29.6, 19.6	30.5, 16.1	0.831
SWS (%)	57.2, 20.5	53.9, 18.6	0.016
REM (%)	13.3, 6.6	15.3, 7.3	<0.001
AI	1.2, 1.9	1.7, 1.8	<0.001

*See Table 1 legend for expansion of abbreviations*.

### Potential Possible Influencing Factors of CA in Children With OSA

By calculating the simple correlation coefficient (Spearman test), we evaluated the correlation strength between the CAI and variables with significant differences. There were positive correlations between CAI and sex, OAHI, AI, OAI, ODI, and REM period. In particular, there were significant negative correlations between the CAI and SWS period (Table 3).

**Table 3:** Simple correlation analysis (Spearman test) of the CAI and potential possible influencing factors in children with OSA.

	**Age**	**Sex**	**OAHI**	**OAI**	**Min Sat**	**ODI**	**SWS**	**REM**	**AI**
r_s_	−0.208	0.11	0.222	0.301	−0.139	0.225	−0.792	0.193	0.318
p	<0.001	0.001	<0.001	<0.001	<0.001	<0.001	0.020	<0.001	<0.001

*See Table 1 legend for expansion of abbreviations*.

First, we used a univariate binary logistic regression model (CAI > 1 = “1,” CAI ≤ 1 = “0”) to examine the factors related to CA (Table 4). Among the factors studied, age, male sex and SWS period were protective factors. The OAHI, AI and OAI were risk factors. REM period was a risk factor. ODI and Min Sat did not significantly affect the CAI.

**Table 4:** Univariate binary logistic regression results: predictors of CA in children with OSA.

	**B**	**OR (95% CI)**	***p*-value**
Age	−0.171	0.843 (0.793~0.895)	<0.001
Sex (boy)	−0.344	0.709 (0.536~0.937)	0.015
OAHI	0.018	1.018 (1.003~1.032)	0.017
OAI	0.068	1.070 (1.036~1.105)	<0.001
Min Sat	−0.005	0.995 (0.979~1.011)	0.515
ODI	0.014	1.014 (1.000~1.029)	0.057
SWS period	−0.009	0.991 (0.985~0.998)	0.014
REM period	0.042	1.042 (1.022–1.063)	<0.001
AI	0.165	1.179(1.079~1.289)	<0.001

*OR, odds ratio; CI, confidence interval*.

Second, the independent significant variables without collinear problems in the univariate binary logistic regression analysis were entered into the multivariate binary logistic regression analysis (Table 5). For each 1% increase in the proportion of REM-stage sleep, the risk of having a CAI >1 increased by 5.3% (OR = 1.053, 95% CI: 1.032~1.076, *p* < 0.001). The CAI increased with the increase in the OAHI. Older age was associated with an increased probability of a CAI ≤ 1, and boys were more likely to be diagnosed with a CAI ≤ 1 than girls.

**Table 5:** Multilevel binary regression results: predictors of a CAI > 1 in children with OSA.

	**B**	**OR (95% CI)**			***p*-value**
Age	−0.179	0.836	0.786	0.890	<0.001
Sex	−0.496	0.609	0.455	0.816	0.001
OAHI	0.023	1.023	1.008	1.039	0.003
REM period	0.052	1.053	1.032	1.076	<0.001

*The results presented in the table represent the final regression model*.

## Discussion

### Distribution Characteristics of CA in Children With OSA

The pathogenesis and clinical manifestations of CA and OA are different. Children with OSA are characterized by OA during nocturnal sleep, which affects their sleep structure and blood oxygen (4, 5), but does CA occur at the same time? In our clinical practice, we found that the results of overnight PSG in children with OSA showed that CA often occurred simultaneously at night. Don seemed to have confirmed this, as he observed a surprising incidence of CA in children with OSA, with an average CAI of 2.5 (16). However, the characteristics of the prevalence and distribution of CA in children with OSA and whether CA has clinical significance have not been elucidated.

In our study, we included 856 children with OSA and found that almost all children with OSA had CA at night, with a CAI > 1 in 50.1% and a CAI > 5 in 2.9%. These indicate that OA and CA coexist in children with OSA, and abnormal CAI is not uncommon in healthy children with OSA.

Central apnea is generally considered a normal physiological phenomenon in healthy infants and children and is considered benign, but there is a lack of research on abnormal CAI thresholds in children with OSA. In this study, the demographic characteristics and sleep breathing parameters of the CAI > 1 group and the CAI ≤ 1 group were further compared, and it was found that the indicators in the two groups is different. This suggests that 1 can roughly be used as a CAI threshold to reflect abnormal central apnea in children with OSA. In clinical practice, when a child with OSA has a CAI > 5, it is recommended to carefully review the underlying disease conditions of the child, paying attention to whether there is a concurrent CSA, and it is recommended to evaluate neuroanatomical abnormalities. For other children with specific underlying medical conditions, further monitoring should be carried out.

### Potential Possible Influencing Factors of CA in Children With OSA

In both our clinic and the already research, we found that central apnea events often occur during sleep in children with OSA. But it is still unknown that the potential influencing factors of central apnea events in children with OSA. Until now, few studies have focused on this topic.

In our study, we observed that the severe OSA group had the highest CAI, and the CAI increased as the OAHI and OAI increased. Regression analysis results further confirmed that increases in OAHI and OAI were risk factors for a CAI > 1, and CAI increased with the severity of OSA; thus, we speculate that the CAI may reflect the severity of OSA in children to some extent. Therefore, in children with OSA, while there may be central apneas which don't merit a diagnosis of central sleep apnea syndrome, central apnea events can certainly be a by product of OSA, higher depending on the severity of OSA. For OSA children with higher CAI, clinicians need to pay attention to the fact that their conditions may be more serious. Especially for children with CAI > 5, we not only need to pay attention to the possibility of comorbidity of OSA and CSAS, but also need to consider the severity of OSA. Genoveva's latest large-sample study also found similar results. They analyzed PSG data of 1,279 children between 1 and 14 years of age over a period of 10 years. They found that patients with OSA had a higher CAI (2.16) than those without OSA (1.17), and this correlation increased with increasing degrees of OA severity (19). The ODI, AI, and Min Sat are important indexes reflecting the severity of OSA. In our study, we also observed that the moderate and severe groups had lower blood oxygen saturation and higher ODIs and AIs than the mild group. Through correlation analysis, we found that there was a positive correlation between the CAI and ODI, AI; and a negative correlation between the CAI and Min Sat. These results further suggest that the CAI can reflect the severity of OSA in children to some extent. However, McLaren summarized normative data from several studies across North America and Europe, and these studies showed that in healthy children, CA was not associated with significant oxygen desaturation, and arousals caused by CA were rare in this population (20). Therefore, we speculated that perhaps the increase in the CAI related to hypoxia only occurred in children with OSA.

Traeger et al. (21) also found that among healthy children between the ages of 2 and 9, older children had fewer CA events. In our study, we similarly found that age to be a protective factor against a CAI >1 in children with OSA. CA decreases as children develop, but the mechanism is uncertain. It is generally believed that CA is more common in younger children, which most likely represents an immaturity of the central nervous system in younger children (16, 22, 23).

In our study, no sex ratio difference was observed among the children with different severities of OSA. Rosemary's study also showed that there was no sex difference in children with OSA; he noted that females with moderate-severe OSA exhibited more internalized problems (anxiety/depression, withdrawal/depression, somatic complaints) (24). However, we found that there was a difference in the sex distribution between the CAI >1 group and the CAI ≤ 1 group. Boys were more likely to have CAI ≤1 than girls. Male sex was a protective factor against CA in children with OSA.

Importantly, the REM period plays an important role in the prediction of CA in OSA children according to our study. We found that the REM sleep period was a risk factor for CA in children with OSA, meaning that the greater the increase in the REM phase in children with OSA, the easier it is for patients to achieve a CAI > 1. Boudewyns et al. (25) also found that in 58 OSA children with CAI ≥ 1, the period of REM was higher than that of total study population (*n* = 98, *P* < 0.01). The mechanisms underlying CA in pediatric OSA are not clear. McLaren explained the different mechanisms contributing to CA, Controller defect, ventilator defect and PaCO2 changes are three primary mechanisms (18). In the REM period, pharyngeal muscle tone decreased, ventilatory control was unstable, and the ventilatory responses to hypoxia and hypercapnia reduced (26). Then PaCO2 increased and a new sleep specific PaCO2 set point was established (27). Central apneas will ensue if the PaCO2 falls below which is usually 2–6 mmHg lower than the eucapnic sleeping level (apnea threshold) (28).

In our study, the correlation analysis showed that there was a significant negative correlation between the CAI and SWS period (rs = −0.792, *p* = 0.02), and the univariate regression analysis showed that a shorter SWS period (OR = 0.991, 95% CI: 0.985~0.998, *P* = 0.014) resulted in a lower CAI value. There was no significant difference between the CAI > 1 group and the CAI ≤ 1 group in the LS period. Therefore, we can conclude that sleep structure and sleep phase play roles in the occurrence of CA in pediatric OSA patients.

The current work has several limitations. The body mass index (BMI) of the children was not taken into account. The association between obesity and CA is unclear. We did not obtain data on PaCO2, as this information was unavailable for many of the cases. The medical history length was not considered in this study. The severity of OSA will increase with the prolongation of the medical history; will CA become increasingly frequent as the medical history length increases in children with OSA? This will be investigated in further research.

As described above, we found that almost all children with OSA experienced CA at night, and a CAI > 1 was more likely to occur in children with OSA, sometimes a CAI >5 may occur in OSA children. The OAHI and REM period were risk factors for CA. Age, male sex were protective factors. CAI could be a supplementary index to reflect the severity of OSA in children. So, in clinical practices, for OSA children with higher CAI, especially CAI > 5, clinicians not only consider the age of children and the possibility of comorbidity of OSA and CSAS, but also to pay attention to the severity of OSA and the sleep construction.

## Data Availability Statement

The original contributions presented in the study are included in the article/supplementary material, further inquiries can be directed to the corresponding author.

## Ethics Statement

This study was approved by the Ethics Committee of the Capital Institute of Pediatrics (SHERLL2019051). In the course of our research, we abided by the strict ethical principles stipulated in the requirements of the committee. Written informed consent to participate in this study was provided by the participants' legal guardian/next of kin.

## Author Contributions

JL collected and analyzed data and drafted the first manuscript. LCh and LCa designed the project and revised the manuscript. GH analyzed data. All authors have read and approved the final version of the manuscript.

## Funding

This work was supported by the CAMS Initiative for Innovative Medicine (2016-I2M-1-008).

## Conflict of Interest

The authors declare that the research was conducted in the absence of any commercial or financial relationships that could be construed as a potential conflict of interest.

## Publisher's Note

All claims expressed in this article are solely those of the authors and do not necessarily represent those of their affiliated organizations, or those of the publisher, the editors and the reviewers. Any product that may be evaluated in this article, or claim that may be made by its manufacturer, is not guaranteed or endorsed by the publisher.

## Abbreviations

AASM: American Academy of Sleep Medicine
AHI: Apnea hypopnea index
AI: arousal index
CA: central apnea
CAI: central apnea index
CI: confidence interval
CSA: central sleep apnea
CSAS: central sleep apnea syndrome
CPAP: continuous positive airway pressure
EEG: electroencephalography
EOG: electrooculogram
EMG: electromyography
ECG: electrocardiogram
ICSD-3: International Classification of Sleep Disorders-3
LS: light seep
OA: obstructive apnea
OAI: Obstructive apnea index
OAHI: obstructive apnea hypopnea index
OSA: obstructive sleep apnea
ODI: oxygen desaturation Index
OR: odds ratio
Min Sat: minimal oxygen saturation
Mean Sat: mean oxygen saturation
PSG: polysomnography
REM: rapid eye movement
SRBD: Sleep-Related Breathing Disorders
SWS: slow-wave sleep.
